# Laccase Engineering: Redox Potential Is Not the Only Activity-Determining Feature in the Metalloproteins

**DOI:** 10.3390/molecules28176209

**Published:** 2023-08-23

**Authors:** Misha Ali, Priyanka Bhardwaj, Hassan Mubarak Ishqi, Mohammad Shahid, Asimul Islam

**Affiliations:** 1 Centre for Interdisciplinary Research in Basic Sciences, Jamia Millia Islamia, New Delhi 110025, India; misha11biochem@gmail.com (M.A.); priyankabhardwaj1409@gmail.com (P.B.); 2 Department of Surgery and Sylvester Comprehensive Cancer Center, Miller School of Medicine, Miami, FL 33136, USA; hxi77@miami.edu; 3 Department of Basic Medical Sciences, College of Medicine, Prince Sattam Bin Abdulaziz University, Al-Kharj 16273, Saudi Arabia

**Keywords:** laccases, rational engineering, electrostatic environment, hydrophobic environment, steric hindrance, orientation of substrate

## Abstract

Laccase, one of the metalloproteins, belongs to the multicopper oxidase family. It oxidizes a wide range of substrates and generates water as a sole by-product. The engineering of laccase is important to broaden their industrial and environmental applications. The general assumption is that the low redox potential of laccases is the principal obstacle, as evidenced by their low activity towards certain substrates. Therefore, the primary goal of engineering laccases is to improve their oxidation capability, thereby increasing their redox potential. Even though some of the determinants of laccase are known, it is still not entirely clear how to enhance its redox potential. However, the laccase active site has additional characteristics that regulate the enzymes’ activity and specificity. These include the electrostatic and hydrophobic environment of the substrate binding pocket, the steric effect at the substrate binding site, and the orientation of the binding substrate with respect to the T1 site of the laccase. In this review, these features of the substrate binding site will be discussed to highlight their importance as a target for future laccase engineering.

## 1. Introduction

Laccases (EC 1.10.3.2, benzenediol:oxygen oxidoreductases) are metalloenzymes that are members of the multicopper oxidase family and are characteristically extracellular monomeric glycoproteins [1,2,3]. Their catalytic function is to oxidize a wide array of phenolic and non-phenolic compounds. This catalytic reaction uses oxygen as an acceptor of electrons, and the only by-product is water [4,5,6]. These enzymes express a broad spectrum of redox potential vs. NHE (normal hydrogen electrode) from +430 to +800 mV, irrespective of their very alike structures [1,6,7,8]. The utilization of oxygen as a final acceptor of electrons without involving expensive co-factors, the generation of water as a lone secondary product, and the promiscuity of these enzymes for a broad variety of organic compounds make them an ideal candidate as biocatalysts for diverse technological purposes [9,10,11]. These purposes include applications in the food industry, chemical synthesis, the paper and pulp industry, and bioremediation [12,13,14,15,16,17]. Despite its wide range of substrate specificity, its low redox potential restricts its applications. Laccases have been reclassified into three categories based on their electrochemical properties: Those with a low, moderate, or high reduction potential at the T1 Cu site [18]. The low reduction potential of plant laccase corresponds to 430 mV vs. NHE [19]. The median range of basidiomycete laccase corresponds to 470 to 710 mV vs. NHE [20,21]. *T. versicolor*, *T. hirsuta*, and all laccases have a high reduction potential range of about 780 mV vs. NHE [19,20]. The reduction potential of laccase was reported by Shleev et al. from a variety of fungal sources, including *Cerrena maxima*, *Coriolopsis fulvocinerea*, *Trametes ochracea*, and *Trametes hirsuta*. The direct redox titration method utilizing mediators was used to calculate the redox potential, which came in the range of 750 to 800 mV vs. NHE [22]. Olbrich et al. found in the site-directed mutagenesis studies that the rise in reduction potential was due to the hydrophobicity of the axial ligand of the T1 Cu, as well as structural alterations in the substrate binding site and more hydrogen bonding. However, the correlation between the T1 Cu redox potential and the oxidation of the substrate is not constant; rather, it varies depending on the substrate. The T1 Cu reduction potential was shown to have a variety of effects on the oxidation of substrates. Although there was no clear association between the T1 Cu redox potential and the kinetic parameters for the oxidation of syringaldazine, a strong correlation was found between the T1 Cu reduction potential and the conversion of substituted phenols, with reduction potentials ranging from 660 to 820 mV [23]. It has a comparatively low redox potential (E^0^ +400 to +800 mV) as compared to other oxidoreductases such as peroxidases [24,25,26]. However, the utilization of mediators is known to broaden their applicability to larger substrates [27,28,29,30,31]. Increasing the redox potential of these enzymes to enhance their application through protein engineering is a challenging task [32,33]. Laccases with remarkable structural similarity and high sequence identity might show relatively contrasting substrate binding sites delimited by different residues. Nonetheless, a similar binding site for the substrate is mostly conserved through a cognate hydrophobic nature, identification characteristics, and spatial arrangement of the binding pockets [34,35]. The diversity of the substrate binding sites and diverse kinetic behavior of laccases with comparable redox potentials indicates the contribution of the amino acid residues delimiting the substrate binding pocket to the oxidizing potential of laccases [7,36]. This led to the conclusion that some other factors, apart from redox potential, might also be responsible for the oxidizing capability of laccase. Therefore, in this review, we aimed to discuss the laccases engineered for modulating other events associated with the binding and oxidation of the putative substrates to highlight their importance in increasing the specificity and activity of enzymes.

## 2. An Overview of the Laccase Structure

### 2.1. Overall Structure of the Laccase

**Figure 1:**
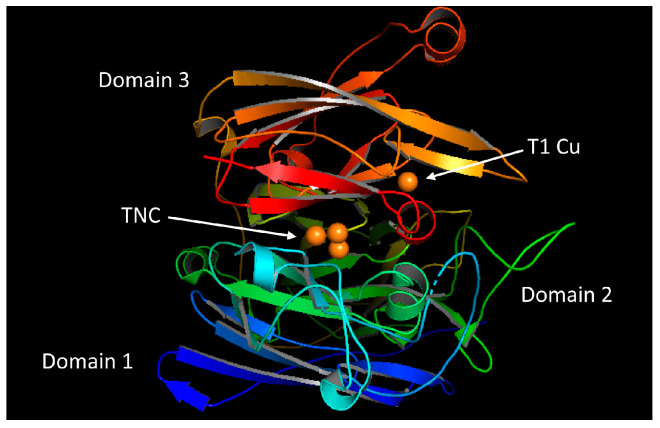
Crystal structure of fungal laccase representing the mononuclear site (T1Cu) and the trinuclear site (TNC).

Laccases exhibit a peculiar fold that consists of three cupredoxin domains, and their active site consists of four copper atoms. These atoms are categorized as type 1 copper (T1Cu) in the T1 site (mononuclear copper center) and a cluster of type 2 and type 3 coppers (T2Cu, T3Cu, and T3’Cu) in the T2/T3 site (trinuclear copper center) as shown in the Figure 1. The distinctive absorbance of the T1 copper site has been found somewhere around 610 nm. This site is responsible for the blue color of the enzyme. The T2 copper cannot be identified by spectrophotometry; however, it generates a distinctive EPR signal [2,37,38]. The absorbance peak can be seen at 330 nm, which is due to the diamagnetic nature of the T3 copper site [39]. Their optical and electro paramagnetic resonance characteristics serve as the main criteria for this classification [2,26,40]. For some laccases, it has been reported that T1 is the primary site where electrons are accepted from reduced substrates. In addition, the catalytic efficiency (k_cat_/K_m_) is influenced by the redox potential of the T1 copper site [2,20,41]. Mononuclear and trinuclear copper centers are localized in the third domain and in between the first and third domains, respectively (Figure 1) [42,43]. The substrate binding pocket is located near the mononuclear copper center and is constituted by the residues of the second and third domains [28,35].

### 2.2. T1 Site

**Figure 2:**
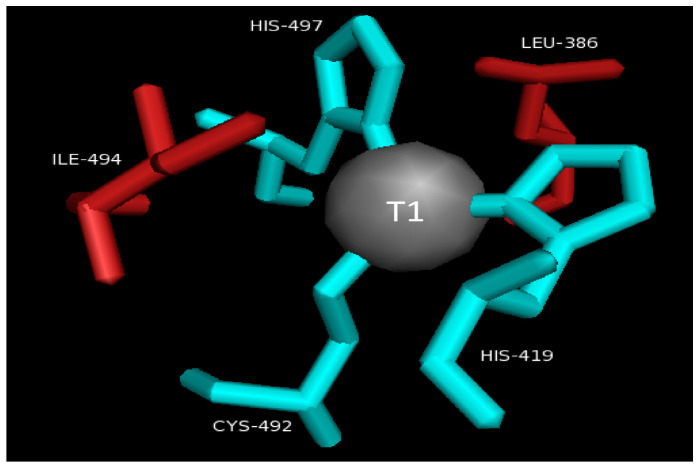
Detailed representation of T1 site.

The T1Cu atom is coordinated by the side chains of one cysteine residue and two histidine residues. The Cu atom and the atoms taking part in its coordination fall in the same plane, while on both sides, side chains of the axial hydrophobic residues are present [44] (Figure 2).

The highly conserved residue isoleucine is situated on one side of T1Cu, while phenylalanine, methionine, or leucine residues could be situated in the opposite direction in all the three-domain laccases that are structurally characterized. The lowest redox potential is of those laccases that contain methionine in their axial position [45,46,47].

### 2.3. T2/T3 Site

**Figure 3:**
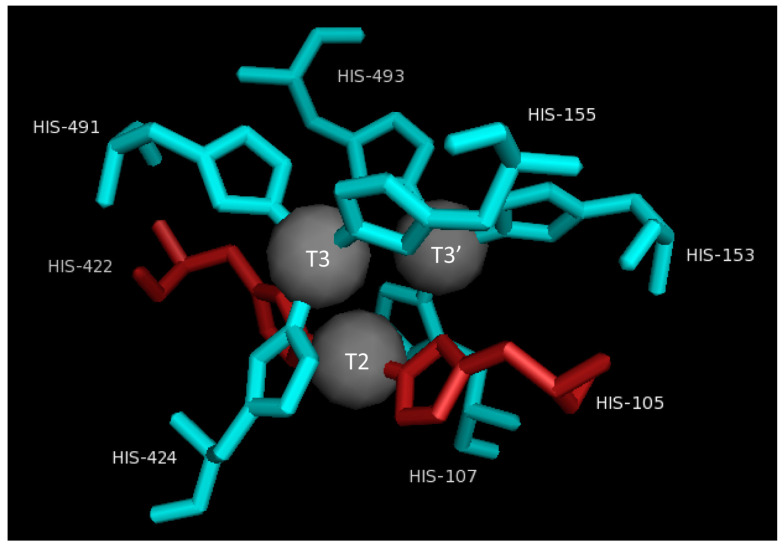
Detailed representation of T2/T3 site.

The T2/T3 site consists of three Cu atoms (T2Cu, T3Cu, and T3’Cu) and eight coordinating histidine residues. The T2Cu atom is coordinated by two histidine residues, and it can be additionally linked by one or two oxygen ligands. This results in the development of a square-planar structure. The T3Cu and T3’Cu atoms are coordinated by six histidine residues. These are coordinated with each other through the oxygen as a ligand shown in Figure 3 [42,48].

**Figure 4:**
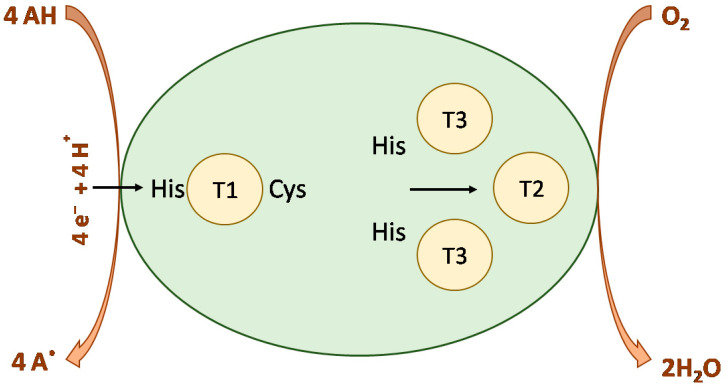
Schematic representation of catalytic cycle of laccase.

The mononuclear T1 site is where the oxidation of the substrate occurs. The electrons are then transferred to the two histidine residues along a cysteine-coordinating T1Cu.The trinuclear site, where the reduction of dioxygen occurs, contains T3 coppers that coordinate with these histidine residues [49,50]. The entire electron transfer process is shown in the Figure 4.

## 3. Characteristics That Determine Activity Other Than the Redox Potential

### 3.1. Electrostatic Environment of the Enzyme Pocket

**Figure 5:**
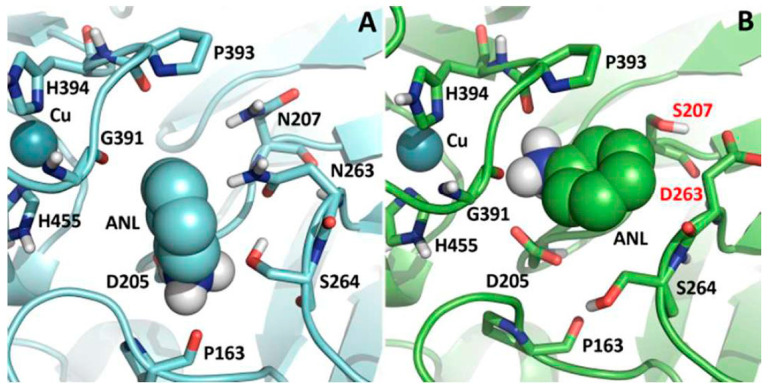
Representation of aniline interaction with (**A**) parent laccase and (**B**) double mutant consists two mutations N207S and N263D [56].

It was first shown by Xu and co-workers that the characteristics of the enzyme pocket determine substrate binding and electron transfer. During the process of substrate oxidation, the presence of an electrostatic environment within the active site of laccase is beneficial to the coordinated transfer of electrons and protons. A significant drop in k_cat_ and increase in K_m_ but no change in the redox potential of T1Cu was observed in *Rhizoctonia solani* (E^0^ = +710 mV) and *Myceliophthora thermophila* (E^0^ = +470 mV) laccase substrate binding pockets with a triple mutation of a tripeptide (LEA and VSG, respectively). The observed changes in substrate docking were attributed to steric and electrostatic hindrances introduced upon mutation [51]. Later on, it was also documented that the substrate forms a hydrogen bond with aspartate (or glutamate) residues placed at the base of the binding pocket, which is completely conserved in fungal laccases [52]. In fact, it was observed in a study that the D205R mutation in POXA1b resulted in a significant waning of catalytic features along with a reduction in its stability. It was revealed through molecular dynamics simulations that the structure of mutated POXA1b is perturbed by the R205 mutation in a greatly conserved region. This leads to a large reorganization of the structure, therefore decreasing both enzymes’ activity and stability [53]. The carboxylate group (D206) is deprotonated at physiological pH (pKa 3.9). Therefore, substrates carrying –NH_2_ and –OH groups are towed inward by the negative charge of this deprotonated carboxylate group and directed to the histidine ligand (H458). During the transfer of electrons to T1Cu through the histidine ligand, the aspartate residue helps in the deprotonation of the substrate. This provides a coordinated electron/proton transfer during the oxidation of phenolic compounds [54]. Monza et al. also demonstrated the importance of a favorable electrostatic environment in the binding and electron/proton abstraction of the substrate. The increased activity obtained by the mutant laccase after directed evolution was rationalized by simulations showing that the mutations P394H and N208S resulted in the stabilization of the oxidized substrate radicle and enzyme complex due to an improvement in the electrostatic environment of the enzyme at the binding site [55]. The recombinant laccase’s activity which has high redox potential was found to be increased upon a double mutation (N207S/N263D) induced in the enzyme. In this double mutant, aniline is sandwiched between negatively charged D205 and D263, thus making it more susceptible to electron and proton transfer for its oxidation. Moreover, the orientation of the binding aniline in the double mutant has changed, with the amine group nearer to the copper atom because of the short side chain of the S207 residue (Figure 5) [56].

A recent study using molecular dynamic simulation demonstrated that electrostatic interactions were the primary forces involved between coniferyl alcohol and laccase. The strong electrostatic interaction of L112 is because of the carboxyl group’s electropositive carbon atom, which is able to electrostatically draw the coniferyl alcohol-attached phenolic hydroxyl group’s negatively charged oxygen atom [57].

### 3.2. Steric Hindrance Due to Bulky Structures

Steric hindrance can also be an activity-determining factor, as bulky substrates may find it difficult to bind at the substrate binding pocket lined by residues with large side chains [58,59]. Tadesse and coworkers investigated the comparative participation of the oxidation–reduction and steric features of presumed substrates in finding their propensity for oxidation in *Trametes villosa* (E^0^ = +790 mV) and *Myceliophthora thermophila* (E^0^ = +470 mV). Even though ∆G^0^ between the substrate and T1 Cu site is a rate-determining reaction step, the results of the investigation show that some of the substituted anilines and phenols did not get oxidized by the enzyme even if they had suitable redox potential. A description was made relating the substrate consumption to the maximum dimension of substituted phenols. Hydrophobic residues (F162, L164, and F265) delimit one of the binding pocket’s sides, while other residues (F332, F337, and P391) are part of the opposite wall. Occupied by the two phenylalanine residues (F332 and F265), which are separated by a distance of 10.8 Å forces involved in interactions between substrate and active site residues of the laccase (excluding the hydrogen atom’s van der Waals radii) form the entrance path of the substrate. A few putative substrates were found to be recalcitrant towards oxidation by *Trametes versicolor* laccase (TvL). The sterically burdened 2,4,6-tri(But)phenol, which measures 11.8 Å (distance between the farthermost methyls of the two ortho-But groups along with van der Waals radii) and 10.5 Å long, had not been oxidized. However, 4-(but)phenol and also 2,4,6-trimethylphenol, which are bulkier but less wide (9.1 Å) than 2,4,6-tri(but)phenol, were completely oxidized [60]. Moldes et al. demonstrated the comparative redox mediating ability of two natural redox mediators on the basis of steric effect. Among the two natural redox mediators, i.e., syringaldehyde and vanillin, the latter was found to be more efficient in the biobleaching of eucalyptus kraft pulp. These two natural mediators are alike in structure and are only distinguished by the number of methoxy groups on their aromatic rings. Vanillin has a single methoxy group, making its phenolic group more sterically accessible than syringaldehyde, which has two methoxy groups in the ortho position with regard to the phenol group [61].

Another very good example supporting steric hindrance as an activity-determining factor was demonstrated by Galli and co-workers. A site-directed mutagenesis strategy was used in TvL to examine how the substitution of the hydrophobic and bulky phenylalanine residues located at the prime positions at the T1 Cu site with small apolar residues that are less hindered, such as alanine, could probably expedite the access of large substrates while simultaneously preserving the non-polar feature of the active site. Oxidation of thymol by wild-type (WT), F162A, and F332A mutants was found to be similar, whereas F265A was comparatively less efficient. The extent of oxidation of thymol by WT and mutants (F162A and F332A) was anticipated to be similar due to the smaller size of the substrate. It has a width of 7.7 Å, which is smaller than the distance of 10.8 Å between the two phenylalanines, F332 and F265, in TvL, which define the entry to the enzyme’s active site. Hence, the alterations done to enlarge the width of the binding pocket entrance should not affect the activity of the mutants (F162A and F332A). While, for the oxidation of 3,5-di-t-bu-phenol, the efficiency of F332A appeared similar to that of WT, F162A exhibited a higher conversion rate as compared to WT, and F265A again showed a lower conversion rate. Docking simulations rationalized the results by showing that F332 is far away from the substrate and does not interact with it; therefore, the F332A mutation did not result in any change in the reactivity. On the other hand, F162 interacts with the substrate and restrains the entrance; hence, the oxidative reactivity of the F162A mutant was mainly due to the increased nearness of the substrate to H458 (one of the T1 Cu ligands) that resulted from relaxation in steric hindrance upon switching of phenylalanine to alanine. Finally, it was observed that F265 is quite far from the active site; therefore, the lowered activity of the F265A mutant is probably due to the disruption of hydrophobic forces between the alanine and the potential substrate [62].

In another study of conformations of the substrate at the active site by docking, it was shown that the bulky planar ring structure of the residues in the catalytic site of laccase creates steric hindrance due to which the substrate finds it difficult to enter the catalytic site of the enzyme [58]. However, the conformations of mediators at the catalytic site revealed that mediators can easily enter the catalytic site and interact with residues without any steric repulsion. The relatively small size of the mediator molecules allows them to easily reach the active site residues through the narrow path. The mediator molecules bind in the vicinity of the T1Cu site [63]. A docking study revealed that, if the distance between amino acids and the mononuclear copper site is less than 25 Å, the percentage of dye decolorization decreases to <20%. On the other hand, if the distance between the amino acids and the mononuclear copper site is >25 Å, the percentage of dye decolorization increases. The proposed mechanism provides a larger space for interaction. When the mononuclear copper sites and the residues are separated by >25 Å, it provides room for efficient interaction between the dye and amino acid, thereby expediting effective electron transfer throughout redox reactions [64]. Srinivasan et al. also pointed towards the possibility of steric hindrance for the low binding scores of some dyes with laccase through a docking study. This is due to the dyes’ large planar ring structure and the side chains of the amino acid proximity to the catalytic site cavity [65,66].

### 3.3. Orientation of Substrate in Binding Site

**Figure 6:**
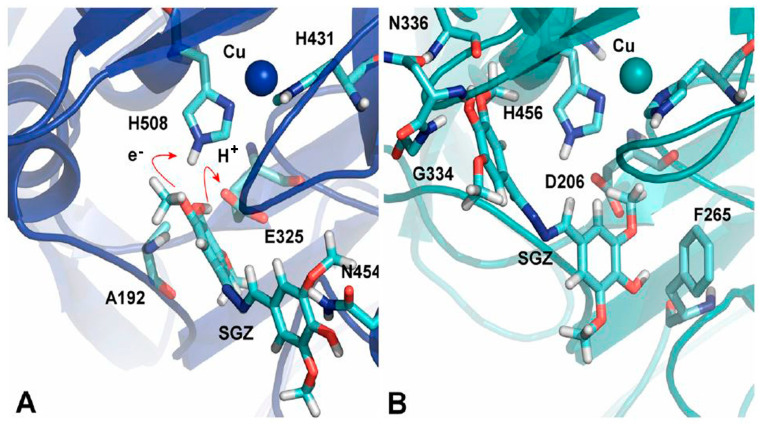
Representation of syringaldehyde binding modes with (**A**) MtL and (**B**) PcL [71].

The crucial element affecting how quickly the substrate oxidizes is the substrate’s orientation in the laccase’s substrate binding pocket. Various site-directed mutagenesis studies have been conducted, which support the importance of the substrate’s orientation in the binding site. Substitution of V148L has resulted in an increased activity of mutant laccase compared to the parental enzyme. The presence of the aromatic ring of Y208 in the vicinity of the leucine side chain may have changed the loop (204–208) conformation at the substrate binding site containing the conserved D205, which interacts with the binding substrate. Conformational changes may have resulted in a favorable orientation of D205 towards the binding substrate, which has increased the reducing substrate’s oxidation, thus increasing the activity of the mutant [67]. Mutation of M168G at the putative substrate binding site of a small low-redox-potential laccase from *Streptomyces coelicolor* resulted in improved k_cat_ (fourfold increase) and K_m_ (tenfold lower), which consequently resulted in around a 40-fold improvement in k_cat_/K_m_ over the WT enzyme. It can be envisioned that substituting the methionine containing the long side chains with smaller residues will increase accessibility to the active site and reduce the distance between the T1Cu site and substrate, therefore enabling the transfer of electrons and enhancing the reaction rate [68]. Xie et al. demonstrated in a study that a change in the binding orientation of syringaldazine in the substrate binding site upon mutation in the laccase resulted in a low oxidation rate [69]. The catalytic efficiency of a high oxidation–reduction potential recombinant laccase was improved for the oxidation of sinapic acid at pH 5.0 by iterative saturating mutagenesis of six residues circumscribing the substrate binding site. Effects of mutations were rationalized by molecular simulations, which revealed that in WT, sinapic acid is bound in an orientation away from the metal center, with its carboxylate group showing interactions with the main chains of G391 and F392 and the -OH group of S387. The mutated variant also showed the same interaction with the substrate with the same residues as in WT, but here F392 is mutated to N392. This F392N mutation rotates the backbone, which results in better binding between the side chains of N392 and substrates containing negative charges and an orientation more buried in the T1Cu site [70]. Lucas and co-workers demonstrated the comparative oxidation of syringaldazine by *Myceliophthora thermophila* laccase (MtL, E^0^T1 = 460 mV) and *Pycnoporus cinnabarinus* laccase (PcL, E^0^T1 = 790 mV) using computational tools. It has been shown earlier that MtL expresses improved kinetics over PcL for the oxidation of syringaldazine in spite of its low redox potential compared to PcL. This was understood by simulations performed to find out the most favorable binding modes of syringaldazine with both laccases. The results showed that binding is more favorable in MtL than PcL, and this favorable binding in MtL was due to the variations in the T1 site. A big loop containing residues 445 to 468, having N454, is present in MtL and holds the substrate in the orientation seen in Figure 6A. On the other hand, PcL involving residues 408 to 416 is a substantially small loop with just eight residues (as compared to 23 in MtL). Another notable variation is the smaller loop with residues 332 to 336 in PcL and 364 to 371 in MtL, which generates a promising condition to anchor the polar –OH group of syringaldazine (Figure 6B). Ultimately, in the active site of the MtL, A192 located at the loop’s end, which is a succession of an α-helix starting with residue 179, helps in anchoring the substrate in a favorable orientation, and this is not present in PcL (Figure 6A) [71]. A molecular dynamics simulation study documented that the hydrogen bonding between 2,6-DMP and D206 as well as the hydrophobic interaction with phenylalanines are substantial interactions responsible for the orientation of the substrate for efficient electron transfer at the T1 site. The H-bonding and hydrophobic interactions of the substrate at the binding site of laccase are disrupted due to mutations at D206 and phenylalanines, which result in the disruption of the proper orientation of the substrate towards the T1 site for electron transfer [72]. In a recent molecular docking study, three compounds, methyl p-coumarate, methyl ferulate, and methyl caffeate, were subjected to biotransformation by *Trametes versicolor* laccase. Despite the substrate similarity, the biotransformation varied greatly. The chemical groups of the phenol ring affect the conformation and orientation of the substrate in the active site. Substrates with the phenol ring, which consist of two groups such as methyl ferulate and methyl caffeate, formed additional Hydrogen bonds with some residues in *Trametes versicolor* laccase–ligand complexes. As a result of the stable complexes, methyl ferulate and methyl caffeate were unable to be transformed [73].

### 3.4. Hydrophobic Environment of the Enzyme Pocket

The hydrophobic milieu of the substrate-binding pocket also plays a crucial role in determining the activity of the laccase [52]. The hydrophobic interaction between the xylidine and laccase through the hydrophobic amino acids that delimit the enzyme’s substrate binding pocket is considered the initial stage of the catalytic pathway [74]. Increased hydrophobic interactions caused by L386W/G417L mutations in *Bacillus subtilis* CotA-laccase were also reported to be responsible for increased enzyme activity [75]. Chen et al. also described that hydrophobic forces are essential for the interaction of laccase with lignin/lignin model compounds [76]. The activity of the double mutant (L386W/G417L) of *Bacillus pumilus* CotA-laccase for the decolorization of dyes was enhanced due to increased hydrophobic interactions between the redox mediator 2,2′-azino-bis(3-ethylbenzothiazoline-6-sulfonic acid) and the catalytic residues [77]. It was also observed that hydrophobic interactions are crucial in maintaining the favorable orientation of the substrate at the binding site [72,78]. The residues F162, L164, F265, F332, and F337 in TvL offer great hydrophobic binding to the subjected substrate to retain it close to the T1 site. The duration of residence of the substrate’s active pose is exploited, which facilitates electron transfer to T1. The presence of hydrophobic amino acids in the enzyme pocket has a significant impact on the enzyme’s activity. The active conformation of 2,6-DMP becomes unstable in the absence of strong hydrophobic interactions [72]. Recently, a molecular dynamics simulation study revealed that the main interactions involved in the binding of nonylphenol and octylphenol with TvL were hydrophobic interactions. Due to the hydrophobic nature of nonylphenol and octylphenol, hydrophobic interaction was the dominant reaction. In the binding between TvL and ligands, the residues responsible for the hydrophobic interactions were expected to have significant roles [79]. In a recent study, it was discovered that the substrate-binding pocket mutations A162V and A458L improved the activity of the laccase mutant for violuric acid and 1-hydroxybenzotriazole.This was attributed to the substrate binding pocket’s increased hydrophobicity [24]. The Lys428 on loop 5 of the *Thermus thermophilus* SG0.5JP17 laccase (lacTT) identifies the substrate as having its side chain oriented towards the substrate entrance. As a result, it was hypothesized that replacing Lys with hydrophobic residues (M428 and L428) might change the conformation of loop 5, whereas replacing Lys with hydrophilic residues (R428 and E428) might not. The K428M mutation, consisting of lacTT, decolorized dye significantly better than the wild type [80].

## 4. Conclusions and Future Prospects

**Figure 7:**
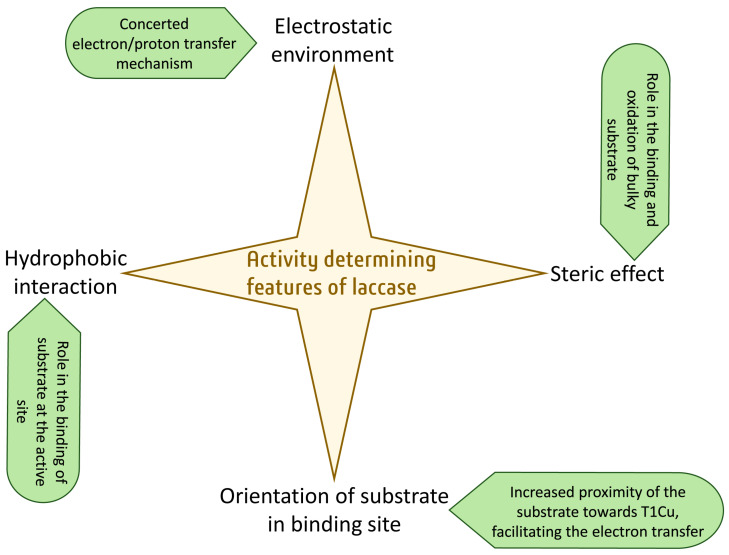
Demonstration of activity-determining features of laccase.

Engineering laccases is of great interest because of their broad range of functions, including industrial and environmental applications. Therefore, the number of laccases has been engineered to increase/enhance their activity and selectivity. But engineering the enzymes with rational methods has always been an appealing approach to saving time and effort. The most logical point that is usually considered for the rational engineering of laccase is to increase the redox potential of T1Cu at the active site. However, increasing the redox potential is a difficult task, as knowledge about increasing the redox potential of T1Cu effectively is still in its infancy. However, there are some other determinants at the active site of laccase that control its activity and can be engineered efficiently (Figure 7). The electrostatic environment at the active site of laccase aids in concerted electron/proton transfer during the oxidation of the substrate. Another feature that regulates the activity of laccase is the steric effect, i.e., whether the substrate is able to find its way to the substrate binding pocket or not. Sometimes, the size of the substrate is so bulky that it is not able to reach the substrate binding site and hence affects the activity. This can be addressed by widening the substrate binding pocket of the laccase. Proper orientation of the bound substrate towards the T1Cu is also very important in determining the activity of laccase. If the bound substrate is more buried towards the T1Cu of the laccase, it facilitates electron transfer from the substrate to T1Cu and hence increases the oxidizing ability of the laccase. Last but not least, the hydrophobic environment at the substrate binding site plays an important role in the binding of the substrate with the enzyme, as the affinity of the enzyme for the substrate is an important feature of enzyme catalysis. Therefore, improving binding affinity of enzyme to the substrate will results in an increased activity. Although a number of features of the active site of the laccase that determine its oxidizing ability have been discovered, extensive research is still required to explore to what extent these features are specific to controlling the activity of the laccase. Moreover, these discussed features should be extensively targeted to engineer the laccases rationally.

## Data Availability

The information that supports the findings of this study is available in this article.
